# Observations on Inguinal Hernia*Read before the Section on Surgery of the Southern Medical Association, at Hattiesburg, Miss., November 12-14, 1911.

**Published:** 1913-03

**Authors:** L. Sexton

**Affiliations:** New Orleans, La.


### TEXAS MEDICAL JOURNAL.

Established July, 1885

F. E. DANIEL, M. D., - - - - Editor, Publisher and Proprieto

Published Monthly.—Subscription, $1.00 a Year.

VOL. XXIX. AUSTIN, MARCH, 1913. NO. 9.

The publisher is not responsible for the views of the contributors.

### Original Articles.

For Texas Medical Journal.

### Observations on Inguinal Hernia.*

BY L. SEXTON, B. S., M. D., NEW ORLEANS, LA.

We can hardly imagine a more inconvenient existence than the truss life of the average hernia patient. This is not altogether the fault of the truss, but is due to the fact that many of the trusses are fitted by drug clerks, who have not the remotest idea of the adaptability of the truss to the hernia. I have seen a truss for the left side worn out on a right-sided hernia. But even when trusses are properly fitted by those trained to sell them in our hot climate, if made of steel, they are subject to the erosions from perspiration, being rusty and soiled.

The man with a hernia is constantly handicapped when wearing a truss with a spring too stiff, and with pressure too great upon the inguinal canal. Many trusses tend to increase the very condition they are made to cure; we refer to the celluloid or hard rubber pad, with a stiff steel spring, that bores its way into the external ring, absorbing all the tissue, making the ring and canal more patulous, rendering the hernia larger and harder to retain after it has descended into the scrotum.

The expense of a truss, particularly those that are made to order, for $10 or $20, are objectionable, because it makes the wearer of such a truss a larger bill during a lifetime than the expense of a hernia operation.

*Read before the Section on Surgery of the Southern Medical Association, at Hattiesburg, Miss., November 12-14, 1911.

The web or elastic truss is unsanitary, and cannot be too severely condemned for its absorption of odors and perspiration and its inability in many instances to retain the hernia.

As said before, the fitting of trusses is too often referred to the druggist or his clerk. In a great majority of cases, many of these drug clerks, without previous training, or ever having seen a hernia, sell and pretend to fit trusses to the first patient that applies, and always the kind of truss which the drug store at that time happens to have in stock.

There is no more fertile source for quackery to exploit the public than in hernia cures and truss-selling advertisements in magazines and newspapers. This is regardless of the fact that many hernias are so large and the patient's labor so strenuous that no kind of truss is sufficient to retain them. These hernias are liable to become strangulated or incarcerated in time, making a serious operation imperative, when a simple one in the beginning might have accomplished a radical cure.

We have often thought how much better it would be for the young patients suffering with hernia to be operated upon by a competent surgeon and under aseptic conditions, rather than living a long hernia life, worried at all times with the inconveniences of wearing a truss.

In our hot climate the disagreeableness of a truss life can hardly be exaggerated, and yet so averse are the general public to any operative procedure that it frequently requires a family meeting (at least in some instances) to get the consent for an operation to relieve even a strangulated hernia.

If the public could only know how few deaths result from herniotomy in properly selected cases, when performed by a competent surgeon, that relapses are the exception and not the rule, we would be able to accomplish much more in this line of surgery than we have ever been able to do in the past. This brings up the question of how many people have hernia. Bull and Coley found "In 33,600 cases of inguinal hernia, males to females, six to one; femoral, male to female, one to two and fourteen-hundredths. Of 22,751 cases, 5554 or 25 per cent were in children under 14 years, while 1381 or 18 per cent of femoral hernias occurred under 14."

Generally speaking, the greater number of cases occur during the active period of life; that is, between 15 and 50 years of age.

While statistics may vary, we may safely assume that one in twenty-five in the male and one in one hundred and fifty among the female patients is about correct.

With such a percentage of curable cases by a simple operation, is it not a pity that this vast multitude of afflicted persons should be worried with wearing trusses of a thousand different makes and kinds during their lifetime?

The question often presents itself to the surgeon as to how early in the child's life may these operations be performed with safety, as it is known that a certain percentage of young people overcome this inherited weakened defect in the abdominal wall as they grow up. Another argument for deferring action is that children do not stand operations very well and the tissue with which the hernial canal is to be closed is tender, and cannot be manipulated with as little impunity as in older patients.

*Diagnosis.*—If from previous inflammation the omentum, mesentery, or intestines, become attached to the hernial sac, or infection, or inflammatory conditions arising from a misfitting truss, such complications might in some instances be mistaken for enlarged and inflamed lymph glands in the inguinal region.

In complete scrotal hernia, you may have to differentiate from hematocele or hydrocele, which is easily done by the introduction of a hypodermic needle or small aspirator.

The cord is felt above the hydrocele at the external ring, but not so in hernia.

Varicocele is usually on the left side, and may empty itself in the recumbent posture; the worm-like feeling of the veins can hardly be mistaken for hernia. Sarcoma and lipoma are very rarely found in the inguinal canal. Light is easily transmitted through hydrocele, while hernia is opaque. So it is to be seen that hernia, as a rule, is very easily diagnosed.

*Predisposing Cause.*—Anything that increases intra-abdominal pressure tends to produce hernia. Any opening through the abdominal walls, whether surgical as in a laparotomy, or natural as the umbilicus, the round ligament, the cord, all leave openings through which intra-abdominal pressure may force out pouches of peritoneum and later hernial contents. Weakened abdominal walls around these openings are inherited in one-third of all hernias. We have known a succession of hernias to occur in grandfather, father and son. So one-third of all hernias are hereditary from this lack of development of muscles around these natural openings in fetal life. A long omentum or mesentery is often the "entering wedge" to the formation of a hernia. The wearing of a truss with a hard pad, as in bubonocele, enlarges the opening in the inguinal canal, from pressure, thus actually predisposing to the formation of hernia, which it is really intended to prevent. So, if a truss life is imperative, the water pad should always be used.

In very many adipose subjects, the fat cells develop between the muscle striæ, which is one of the reasons for hernia being so common in very fleshy persons. Habitual constipation, gaseous indigestion, chronic bronchitis or whooping cough, enlarged prostate, stricture and bladder stones, falling from a great distance, straining and heavy weights, violent sneezing or coughing are among some of the most common predisposing causes. All heavy work, as in farm labor, running and jumping, as practiced by school boys, the blowing of wind instruments; in fact, as said before, anything increasing intra-abdominal pressure predisposes to the formation of hernia.

*Strangulation.*—It is when the hernial sac is large, the neck constricted, and a large loop of intestine descends into the sac, that this constriction takes place, the return flow of blood is obstructed in the bowel, edenia, and inflammation supervening, bringing about pressure which retards the return flow of blood, causing the strangulation, which, if not relieved under the anesthesia in the Trendelenburg position, necessitates an immediate operation. Nearly all hernias are reducible when they are just forming, and only become incarcerated or impossible of reduction after some inflammation or infection has taken place from bad-fitting truss or other cause, or after becoming strangulated. Very many apparently irreducible hernias may be returned to the abdomen by application of ice bags for a considerable time, followed by a general anesthesia in the customary Trendelenburg position. As a matter of course, it is always best in these cases to operate. I am only speaking of this method of reduction of hernia when the patient will not submit to the radical operation under any circumstances.

It is important to remember, clinically, that the posterior wall of some hernial sacs may be made of the continuation of the bladder, cecum or sigmoid.; so, in opening the hernial sac in the sliding variety of hernia, bear these organs in mind. Double sacs of peritoneum, like the hour-glass sac, are rare in occurrence. The contents of the sac are more variable than the walls; for instance, the anterior wall of the bladder, in bese and bulging cases, is occasionally found in the hernial sac; as is also the appendix, ovary and tubes; in fact, in many large neglected hernias, any of the contents of the abdomen may be found in the unusually enlarged sacs. The sac descends behind the epigastric vessels in direct inguinal hernia. The cord in front or to the side of the sac may be found in direct hernia.

Nearly every successfully operated case brings another patient.

Every unsuccessful infected or recurring hernia retards surgery in general and hernia operations in particular. Every recurring case is not necessarily a failure on the surgeon's part, but a neglect of the patient to avoid employment that predisposed to hernia in the first instance. Many abdominal walls are inherently weak about the inguinal canal; usually after hernia operations it is best for the subject to follow some light work; this is particularly true of old and flabby patients.

As said before, where the hernia is complete and the inguinal ring so large that it cannot be retained by any ordinary truss, the surest cure for such a case is assured by castration, but it is necessary to get the patient's consent, as a matter of course, before he takes the anesthetic, provided it becomes necessary to go this far in order to insure no returns.

The best fitting truss may retain the small and easily reducible hernias; so, many patients prefer this plan to a more radical operation; but there is the constant risk of strangulation at some future time or place when proper surgical assistance is not obtainable. This is the fatal ending of many old hernia patients. Obesity in subjects should be reduced by walking exercise, massage, and the simplest diet. For breakfast, a small cup of tea, a tablespoonful of milk, one lump of sugar, a slice of toast bread or cracker. Dinner, one slice of bread and cracker, small piece of lean meat or chicken, one egg and vegetable, salad or apple, glass of skimmed milk, sour wine, or lemon water. Supper, one-fourth of small chicken, boiled or poached egg, slice of toast bread or cracker; any seasonable acid fruit may be taken in moderation between these meals, but as little fluid as possible should be imbibed. This should preferably be light sour wine or lemon water. A very fat patient should walk from five to ten miles a day, varying the meals suggested above according to his own taste, but never drinking any liquids during meal time. Under this diet and exercise, the patient will lose from five to ten pounds per week. Cold or hot baths with friction rubs may be added to this regime. All adipose patients should undergo this reduction treatment until the abdominal walls are largely rid of fat, as our commonest failures and recurrences of hernia operations are in our obese patients. Constipation and gaseous indigestion, if present in any hernia case, should be corrected before any operation is undertaken. Always eliminate all predisposing causes of hernia before any radical cure is attempted.

In all operations for strangulated hernia, make a radical cure unless infection or gangrene render drainage imperative. If trivial heart or kidney complications render general anesthesia inadvisable, local analgesia or spinal puncture may be admissible in specially adapted cases. However, nearly all operable cases can safely take ether. Special preparation of the patient by abstaining from food, and having the bowels properly emptied and the operative field properly prepared, so that infection and vomiting is improbable makes the prospect of a radical cure much better than when any of these details are neglected.

Without describing any particular operation for hernia with which you are all familiar, we will simply remark, in passing, that, after the usual three-inch incision is made over the hernial tumor and extended down to the sac, the fat is best gotten rid of by a finger dissection with damp gauze.

The sac is usually found just above the cord and should be separated from it by a gauze or blunt dissection, without injury to the spermatic cord with little trauma to the soft parts. The lower end of the sac is held up and opened. Any intestines confined in the sac are pushed back, while omentum, if found, is cut away, after being transfixed and ligatured; the sac is then followed up into the abdominal cavity, securely tied with a double catgut ligature. After the distil end of the sac has been cut away, the stump is dropped back into the abdomen.

The neglect of any of these simple details are the causes of recurrences is our only excuse for mentioning them. The transversalis and internal oblique muscles are then sewed to the ledge of Poupart's ligament by four interrupted kangaroo tendon, or catgut sutures.

The epigastric vessels are avoided by inserting the needle from within outwards through Poupart's ligament. The edges of the would should be closely approximated by careful suturing or Mischell clamps. The wound may be either hermetically sealed with a collodion and cotton dressing, or with a large pad of sterile gauze, around which a figure of eight bandage is so placed as to exclude the possibility of any infection after the operation is finished. As to the mode or plan of operation, we personally prefer the Bassini, as it transplants the cord, which is really the guide to the hernial sac, from the inguinal canal to a new position under the external abdominal muscle, leaving less probability of recurrence than in some of the other more complicated operations. The Bassini is easy to perform by lifting the cord after it is separated from the sac out of the way by a soft piece of gauze being placed under it, and in order to snugly close the internal ring putting two sutures of kangaroo tendon or catgut above the cord and three or four interrupted sutures underneath it will render the likelihood of a recurrence very remote.

The cord is thus transplanted to a new bed just under the external abdominal muscle. Many surgeons are just as successful with Ferguson's or Andrew's operation, which has the advantage of being easier to perform, disturbing the cord less and not so likely to be followed by atrophy of the cord or testicle.

Either one of these procedures are about equally effective in bringing about a cure which, in the great majority of cases, is permanent, provided no great intra-abdominal pressure or heavy work is attempted by the patient in the near future. Some complete hernias are so large, remaining in the scrotum, that it is impossible to hold them up by any ordinary truss. To this class of patients, a successful operation is a great boon. But, in order to make a radical cure in some of these cases, it may be necessary to perform a castration in order to make the operation truly successful and to prevent any likelihood of a recurrence.

There is no class of surgical work that requires asepsis more than hernia operations. The final success with no recurrences depends upon the operation being properly performed and the wound not becoming infected. The operation should be done with gloves and with as little bruising and handling of the tissue as possible, with every other known aseptic precaution both before and during the operation.

Carrying out the operative suggestions made above, we have had a series of twenty cases all resulting in a cure, with no complications except an infection in one of the cases, which necessitated a second operation. We have not heard of any serious results from an operation from hernia, though it is performed many times daily in the Charity Hospital and other institutions in New Orleans. The only serious consequences or results which have followed hernia operations have been in those cases in which strangulation had taken place, gangrene followed, necessitating a very serious operation of resecting the bowels, where a very simple or trivial operation would have relieved the case had it been performed at the proper time. As soon as the public are educated to the importance and relief of earlier operations in hernia, as in appendicitis, the better result for the patient and reputation for the surgeon.

---

Change of Date.—The Texas State Medical Association will meet at San Antonio, May 6-9, instead of 9-11, as heretofore announced.

